# Index finger pointing posture in a patient with drug-induced foot tremor

**DOI:** 10.1016/j.prdoa.2021.100111

**Published:** 2021-10-07

**Authors:** Gohei Yamada, Takanari Toyoda, Eiichi Katada, Noriyuki Matsukawa

**Affiliations:** a Department of Neurology, Nagoya City University West Medical Center, Aichi, Japan; b Department of Neurology, Nagoya City University Graduate School of Medical Sciences, Aichi, Japan

**Keywords:** Drug-induced movement disorder, Index finger pointing posture, Serotonin toxicity, Paroxetine, Tramadol

## Abstract

A 41-year-old woman who had been taking paroxetine began taking tramadol for bilateral ankle pain. A few days later, the patient presented acutely with both feet tremors. During a mental arithmetic task, index-finger pointing posture briefly appeared on the left side. Co-administration of paroxetine and tramadol increases the risk of serotonin toxicity. This is the first reported case of index-finger pointing posture which was associated with serotonin toxicity.

## Highlights

• Drug-induced index finger pointing posture has not been reported.
• A 41-year-old patient began taking tramadol in addition to paroxetine.
• The patient presented with acute foot tremor and brief index-finger pointing posture.
• Serotonin toxicity related movement disorder has a broad clinical spectrum.

## 1 Introduction

Index-finger pointing posture is an unusual neurological manifestation commonly observed in patients with craniocervical dystonia, Parkinson’s disease, essential tremor, and epilepsy [1], [2]. The prevalence of index-finger pointing posture in diverse neurological disorders remains unclear. Herein, we report a case of foot tremor with index-finger pointing posture. The cause of foot tremor and index-finger pointing posture was presumed to be the combined use of paroxetine and tramadol. To the best of our knowledge, this is the first reported case of drug-induced index-finger pointing posture.

## 2 Case report

A 41-year-old woman with a history of depression developed intermittent shaking of both feet. She had been taking paroxetine (50 mg/day) for more than ten years. A few days before the onset of the tremor, the patient began taking tramadol (150 mg/day) for bilateral ankle pain. The pain was well controlled. The patient had no complaints of fatigue, agitation, or anxiety. Her vital signs were as follows: blood pressure, 136/92 mmHg; heart rate, 103 bpm; and body temperature, 36.9 °C. Neurological examination was remarkable for foot tremor (Supplementary Video) and generalized hyperreflexia. When the patient attempted to maintain her foot in plantar flexion, the tremor was exacerbated. Quick holding of her arms outstretched did not disrupt the foot tremor. When the patient was holding her unilateral foot off the ground, grounding with her whole sole, or lying in the supine position, tremor did not appear. Ankle clonus was absent. Muscle weakness, hypertonicity, ataxia, and sensory impairment in the upper and lower extremities were not observed. Since the onset of tremor depended on the posture of the foot, essential or enhanced physiological tremor was considered. When the patient was performing a mental arithmetic task, the left index-finger suddenly extended, and a tremulous movement immediately appeared (Video). During the task, the foot tremor persisted. In laboratory tests, hepatic function, renal function, thyroid function, blood glucose, and serum concentrations of sodium, calcium, and magnesium were within the normal range. Magnetic resonance imaging (MRI) of the brain revealed no causative lesion. Paroxetine is a selective serotonin reuptake inhibitor. Although tramadol is an opioid, it blocks serotonin uptake by inhibiting the serotonin transporter and acts as a direct serotonin-releasing hormone. Paroxetine is a selective serotonin reuptake inhibitor. Hence, the co-administration of paroxetine and tramadol increased the risk of serotonin toxicity. The patient fulfilled the Hunter Serotonin Toxicity Criteria with tremor and hyperreflexia [3]. Enhanced physiological tremor represents a phenotype of drug-induced movement disorders. Because depressive symptoms were absent for several years, paroxetine was discontinued. One week after discontinuation of paroxetine, foot tremor, index-finger pointing posture under a mental arithmetic task, and generalized hyperreflexia had disappeared (Video). Her mental status remained unchanged. Based on these clinical findings, the patient was diagnosed with a serotonin toxicity-related movement disorder. The tremor has not relapsed for over three years.

## 3 Discussion

Drug-induced index-finger pointing posture is a unique finding in the present case. A “pointing gun” dystonic posture, which is observed in patients with progressive supranuclear palsy, represents a neurological sign similar to the index finger pointing posture; however, it is characterized by an extended thumb and index finger with the other fingers flexed [4]. In a previous study, index-finger pointing posture was observed during walking in non-psychogenic movement disorders, including craniocervical dystonia, Parkinson’s disease, and essential tremor [2]. This symptom is likely to be a mild form of hand dystonia. The distinctive feature in the present case was that index-finger pointing posture appeared not during walking, but when the patient performed a mental arithmetic task. However, it is well known that dystonia is induced by various tasks, including mental arithmetic tasks. Index-finger pointing posture in the present case could be a dystonic posture.

MRI of the brain did not show any lesion, and electroencephalography was not performed. However, the origin of the index-finger pointing posture seemed to be the central nervous system, because abnormal finger movement was induced by a mental arithmetic task and strictly localized in the left index finger. The central nervous origin is also supported by the high prevalence of index-finger pointing posture in patients with localization-related epilepsy during generalized convulsions [5]. In a positron emission tomographic scan study, communicative pointing posture was associated with the activation of a small area in the medial prefrontal cortex and the temporoparietal junction [6]. As both paroxetine and tramadol inhibit serotonin uptake, a serotonergic mechanism is likely involved in the present case. The increased concentration of serotonin in the synaptic junction might induce hyperexcitability in the medial prefrontal cortex and the temporoparietal junction, triggering this unique finger movement. Index-finger pointing posture is a mild and brief neurological sign that may be overlooked in patients with outstanding foot tremor, such as in the present case. Although tremor, myoclonus, and dystonia are induced by serotonergic drugs, abnormal single finger movements have not been reported. The present case highlights the broad clinical spectrum of serotonin toxicity-related movement disorders.

## 4 Funding disclosure

This research did not receive any specific grant from funding agencies in the public, commercial, or not-for-profit sectors.

## 5 Patient consent form

We retained patient consent form to use case details and video tape of movement disorder.

## CRediT authorship contribution statement

**Gohei Yamada:** Conceptualization, Writing - original draft. **Takanari Toyoda:** Writing - review & editing. **Eiichi Katada:** Writing - review & editing. **Noriyuki Matsukawa:** Writing - review & editing.

## Declaration of Competing Interest

The authors declare that they have no known competing financial interests or personal relationships that could have appeared to influence the work reported in this paper.
